# Social mechanisms for integrating community cats into community governance in urban China

**DOI:** 10.1371/journal.pone.0308120

**Published:** 2024-08-14

**Authors:** Di Wu, Jintu Gu

**Affiliations:** Department of Sociology, Hohai University, Nanjing, China; University of Minnesota, UNITED STATES OF AMERICA

## Abstract

The presence of community cats roaming freely in urban spaces has caused considerable controversy. This is because the management and care of community cats have yet to become part of urban community governance. This research analyzes the process and mechanism of integrating community cats into urban community governance from the interaction between community cats and urban residents. Data were collected through participatory observation and unstructured interviews. Drawing upon the analytical framework of ’Norms, Trust, and Networks’ derived from social capital theory, this research reveals that social norms and trust activate the social capital of the community, fostering a social network with ’community cats’ at its core. More importantly, this social network extends the scope of care from the community cat to other members of the community. This research defines this mechanism as ’care extension.’ This not only fosters a neighborly relationship between residents and community cats that goes beyond mere ecological interactions, but also helps foster a compassionate and harmonious multi-species urban community.

## Introduction

Urbanization is not de-naturalization but rather a change in the external environment that people and non-human animals experience together. However, the relationship between humans and non-human animals has yet to become a public matter of urban community governance in urban China. Non-human animals are marginalized in community governance. Urban community governance has long focused mainly on the needs and interests of human beings, neglecting the status and rights of non-human animals. This has not only led to many challenges to the survival of non-human animals in cities, such as loss of habitat, lack of food, traffic injuries and deaths, human interference, but it has also triggered conflicts in the perceptions and attitudes of residents towards animals [1]. Cats living freely in urban spaces have caused intense social debate [2]. Some believe that these cats pose a serious threat to wildlife, especially birds and small mammals. Others argue that these cats are part of the urban ecosystem. They point out that these cats not only control rat populations, but also provide emotional support for many residents [3]. Previous researches have named these cats from different perspectives, such as ’outdoor cat’, ’stray cat’ and ’free-roaming cat’ [4–6]. Some researchers have named these cats ’community cats’ based on the responsibility of residents to care for them. ’Community cats’ are cats that live in urban communities and are cared for and managed by community residents [7]. Although they usually do not have regular owners, they are able to live healthy in urban neighborhoods with the feeding, care and management of community residents [8].

In order to reduce the risk of community cats to wildlife, while respecting and safeguarding their rights and well-being, it is necessary to develop a humane management plan. Existing research has found that people, as members of the urban ecology, can take action to positively influence the community cat population [9, 10]. Some residents have formed organizations to participate actively in caring for and managing community cats. They not only provide healthy and safe food for community cats but also provide them with medical assistance and implement TNR programs [11]. This meets the survival needs of community cats and reduces the number of community cats [12]. At the same time, the establishment of a community governance system to manage community cats can also effectively solve the problems caused by community cats, such as noise nuisance and disease transmission [13]. More importantly, the care and management of community cats by community residents can promote interaction and cooperation among residents and create a more harmonious community [14]. However, the debate on ’how to treat community cats’ has become increasingly heated among urban residents and has even evolved into a violent conflict between groups with different opinions. This has not only caused a breakdown in social relations but has also reduced the public space for rational discussion of the topic. Driven by such extreme emotions, there are also vicious incidents, such as animal cruelty, which is against modern civilization and social morality. Therefore, it is particularly important to integrate community cats into community governance. The resident-animal relationship is closely related to whether the urban community can be harmonious and whether public safety can be guaranteed.

The Animal Civilization Construction Association(ACCA), located in the S community in Hangzhou, has developed the country’s first Community Animal Civilization Convention on the care and management of community animals. This convention encourages community residents to treat all animals civilly, especially community cats. On this basis, the ACCA has set up a consultation platform for residents to resolve animal-related disputes promptly. With the ACCA’s efforts, the S community has successfully incorporated community cats into community governance and become a model community for harmonious coexistence between people and community cats. Currently, the experience of the S community has been successfully replicated in six neighboring communities. This research will take the ACCA as the research object to analyze the social mechanism of integrating community cats into community governance and explore the significance of this practice for multi-species cities.

Sociologist Park categorized relationships between species into symbiosis and society. It can be seen that there are two different kinds of relationships between humans and animals: ecological and social relationship. Ecological relationships refer to human-animal interactions in the natural environment, such as predation, competition, and symbiosis. Social relationships refer to the interactions between humans and animals in daily life, such as animal breeding and animal rescue. However, previous researches have focused only on the ecological relationship between residents and community cats, ignoring the social relationship between the two. These researches paid attention to the connection between community cats and other species and the natural environment, and regarded community cats as an invasive species that triggers urban ecological problems [15, 16]. On the one hand, community cats threaten wildlife regarding population size and reproductive fecundity [17, 18]. On the other hand, community cats are disease vectors for humans, other domestic animals, and wildlife [19]. Based on concerns about ecological relationships, these researches advocate strict management of community cats. For example, Xu advocated lethal management and suggested increasing public support for lethal management for community cats [20].

In human-animal social relations, Humans, as members of the urban ecology, can take action to impact community cat populations positively. Nordensvard calls for the re-establishment of social citizenship in order to consider animals as an integral part of social governance policies [21]. Donaldson categorizes non-human animals as wild, domesticated, and marginal. Marginal animals are non-domesticated animals that have adapted to live near human communities. He further stated that these marginalized animals living in urban communities should be given resident status [22]. Based on the conceptual framework of ’One Health’, the researcher points out the importance of considering human factors in the management of community cats [23]. By promoting public policies, community cats can be better managed to ensure optimal health for humans, cats and wildlife. The researcher further points out that animal welfare experts, ecological protectors, residents, and governments can combine their skills and resources through cooperation based on consensus and trust to ensure community cat welfare and resolve their negative ecological and social impacts [9]. However, some researchers have found that there are growing concerns related to animal welfare in many cities, but the urban policy agenda does not focus on it [24]. Therefore, it is crucial to examine how community cats become the focal point of urban governance. Unfortunately, these researches have yet to elucidate specifically how animals are incorporated into people’s relational networks. That is, it is not clear how human-animal interactions are intertwined with human-human interactions.

**Fig 1:**
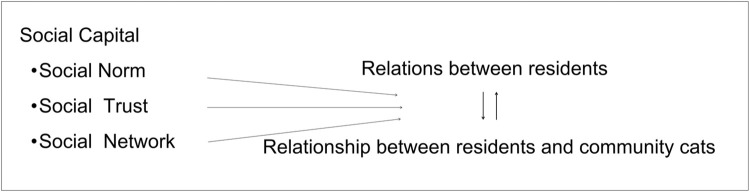
The analytical framework.

In community governance research, social capital theory is considered by most researchers to be the most widely used theoretical tool. Bourdieu defines social capital as an institutionalized network of connections with actual or potential resources [25]. Researchers emphasize the three elements of social capital: trust, norms, and networks [26, 27]. Social trust is conducive to the establishment of emotional ties and mutual support between residents and neighborhood committees. This is beneficial to facilitate the common pursuit of public interests by all parties, thus reducing the cost of community governance [28]. Relationship networks provide structural support for community governance and promote collective action based on a consensus [29]. The tighter the network of relationships, the more conducive it is to mutual assistance and cooperation. This is also conducive to realizing autonomous cooperation and coordination instead of the original administrative task-centered management mechanism [30, 31]. Reciprocity norms that emphasize a sense of contract, cooperation for mutual benefit, and honesty and trustworthiness provide a binding institutional mechanism for community governance. This also reduces the cost of monitoring cooperation, avoids opportunistic behavior, and thus enhances the level of cooperation among community residents [32]. Social capital theory provides a powerful theoretical framework for analyzing the process of integrating community cats into community governance. Establishing effective social networks, setting norms, and building trust are important measures to ensure that community cats are well managed and cared for. The analytical framework of ’norms, trust, and networks’ helps to understand the key factors in managing community cats and analyze the complexity and diversity of the relationship between residents and community cats. See Fig 1 Analytical Framework.

## Materials and methods

### Case briefs

Established in 2009, the S community in Hangzhou has 29 residences and more than 12,000 people. As with most urban communities, the S Community faces the prominent problem of conflict between residents and community cats. On the one hand, the excessive number of community cats has aggravated the residents’ concern about community health issues and affected people’s quality of life. On the other hand, some residents do not understand the behavior of caring for and feeding community cats and view cat caregivers as troublemakers. They often complain to express dissatisfaction with the cat caregivers. In the face of conflicts between residents and community cats, the community council initially only adopted poster campaigns to call on residents to care for community cats. However, these declarations remain only in words, and many residents are still in a state of ’knowing and acting separately. ’ They will not actively support or participate in the care and management of community cats. Meanwhile, there were few resources in the S-community and no platform for cooperation between the parties. At that time, the community often adopted the rough ’catch and release’ method, which involves capturing community cats and then releasing them into other areas. This did not effectively reduce the number of cats in the community. It also raised concerns about the welfare of the relocated community cats and the impact on the ecosystem of the new area.

**Fig 2:**
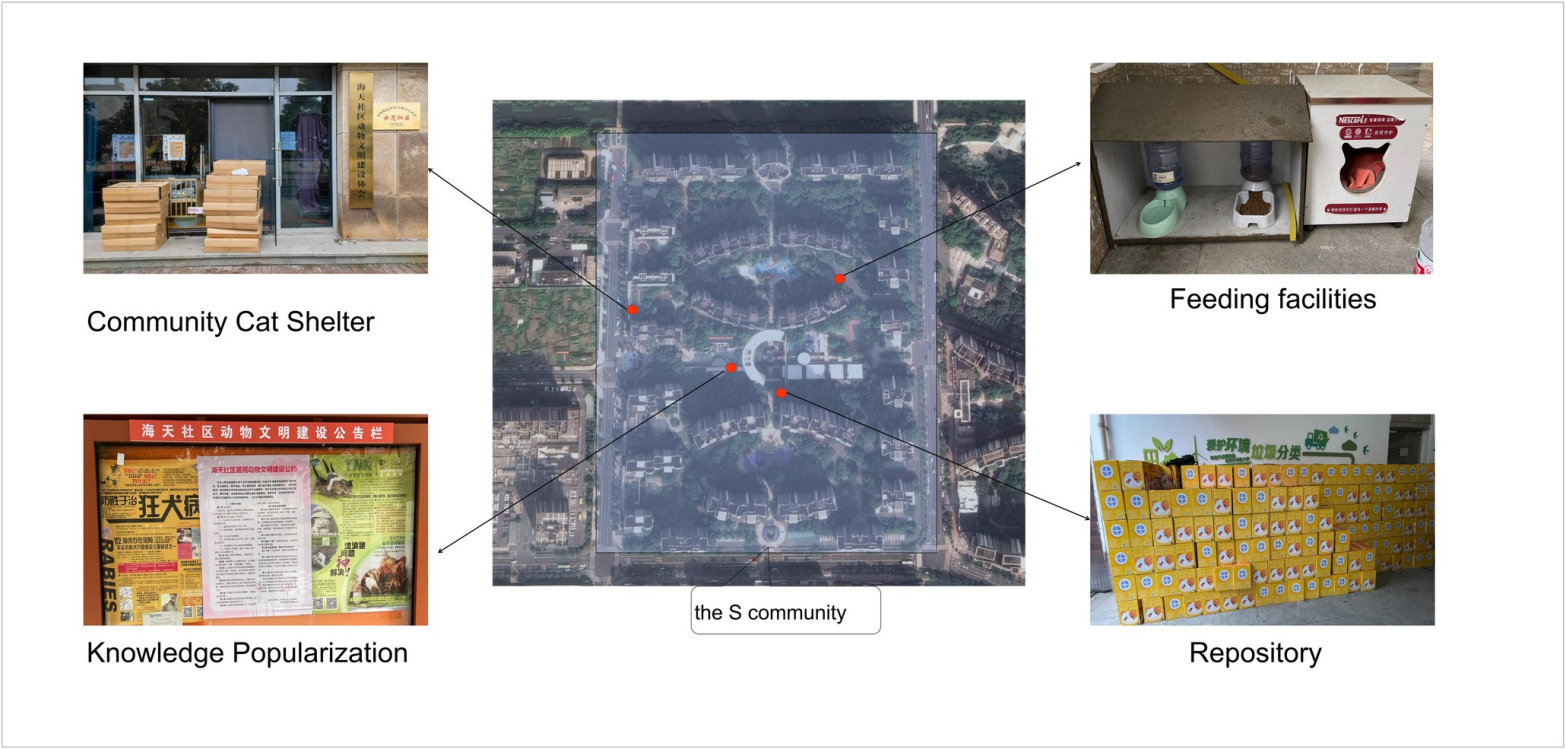
Implementation of ACCA’s work.

In 2002, in order to resolve the conflicts guided by community cats and to safeguard the quality of life of community cats, the cat caregivers in the S community spontaneously set up the Animal Civilization Construction Association(ACCA). The Association first invited animal protection experts to hold lectures in the community to promote scientific and civilized management of community cats. This provided the initial program for residents to coexist with community cats. On this basis, the association took the initiative to undertake the care and management of community cats. First, they adopt the prevailing TNR method to avoid excessive growth in the number of community cats. Secondly, they ensure the health and safety of community cats through feeding, disease treatment, and vaccination. Thirdly, in order to promote the adoption of community cats, they not only formulate socialization training for young cats but also conduct a comprehensive assessment of adopters to avoid incidents of cat abuse and abandonment. Finally, they also create a community environment conducive to the survival of community cats through extensive publicity and education. ACCA’s performance can be seen in Fig 2.

**Fig 3:**
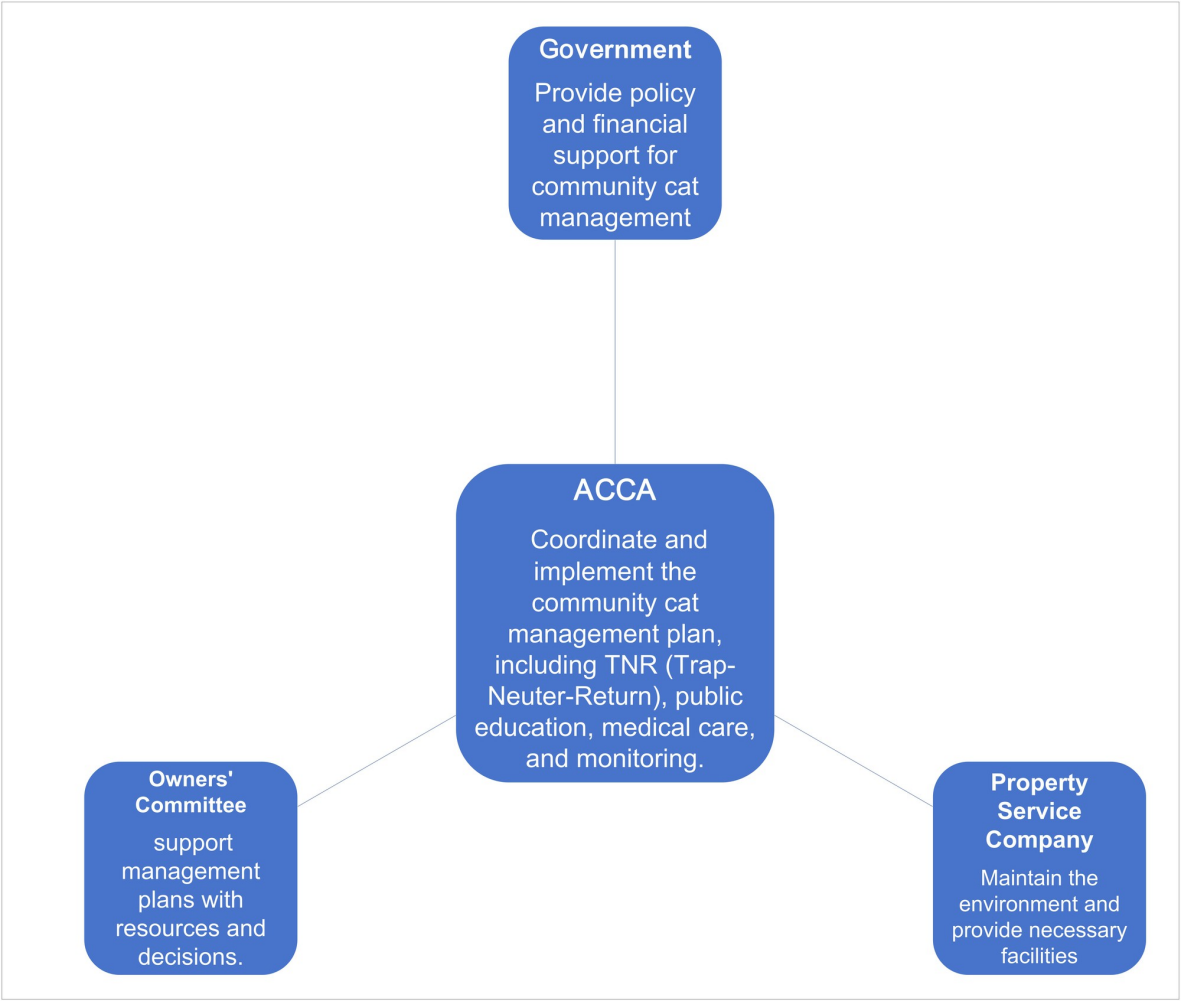
The governance system led by ACCA for community cats.

With the work of the association, the community cats have successfully integrated into the governance system of the local community. And most of the residents changed their attitude of resistance or indifference and began to treat the community cats as ’neighbors’ sharing the living space. Fig 3 illustrates the governance system led by ACCA for community cats. The number of community cats has gradually stabilized, decreasing from 59 in 2019 to 25 in 2023.

**Fig 4:**
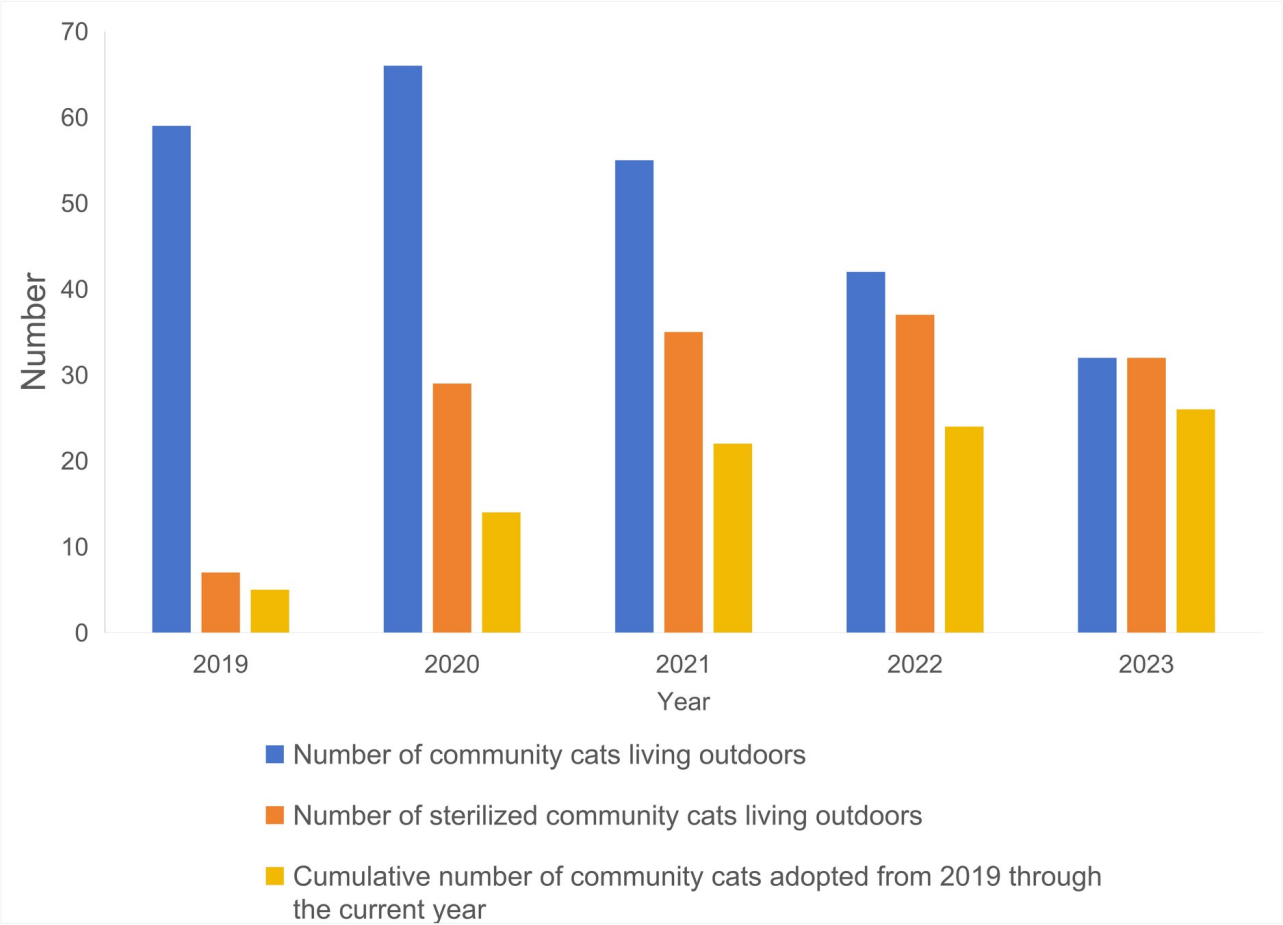
The number of community cats.

At the same time, community cats’ survival quality has significantly improved. Through the electronic profile, every community cat receives safe and fulfilling food and effective medical treatment. The experience of the S community has now been replicated in six neighboring communities and has received praise from several official Chinese media. As of 2023, all community cats within the boundaries of the S community have been sterilized. The cumulative number of adopted community cats is more than 30. Fig 4 shows the trend of the number of community cats within the S community.

### Study design

The researcher first entered the S community in May 2023. In line with the action research paradigm, the researcher was deeply involved in the work of ACCA. The researcher served as the vice president of the association, with a focus on advocacy and the organization of academic seminars. Action research implies that the researcher is no longer independent of the research subject, but works with the subject to identify, analyze, and solve problems in action [33]. This facilitates the translation of knowledge into action, identifies barriers to action, and ultimately promotes changes in consciousness and action [34]. Although the knowledge gained from action research is contextual and localized, it can be used to improve the theory of action research and draw general conclusions through collaborative research with other scholars.

The researcher collected information through participatory observation and unstructured interviews. Participatory observation mainly records the association’s work, the interaction process between community residents and community cats, and changes in the community environment. The period of participant observation for this research was from May to September 2023. Through participatory observation, the researcher was able to record how members of the association cooperated with each other to manage the community cats. This method is beneficial in capturing the details of daily management, such as negotiation, division of labor, and cooperation among different members. In addition, the researcher observed and recorded the interaction between community residents and community cats. This contributes to a better understanding of residents’ attitudes, behaviors, and emotional responses to community cats. For example, the details of residents’ actions in feeding and rescuing community cats provide rich first-hand information for this research.

### Sampling

On the basis of participatory observation, this research selected respondents from members of ACCA through snowball sampling. Snowball sampling means that the first respondents are first randomly selected. The first group of respondents then recommends respondents who possess the overall characteristics of the research objectives [35]. Snowball sampling is beneficial in obtaining information from key people efficiently compared to other sampling methods [36]. The data saturation method was used to determine the number of respondents [37]. The data saturation method means that the number of respondents is continuously increased until the information provided by the respondents begins to be repeated. Based on this method, a total of 23 respondents were selected for this research.

The information on respondents for the unstructured interviews can be found in Table 1. The gender ratio of these respondents was relatively balanced, comprising 12 females and 11 males. The respondents have diverse roles, including two presidents, four vice-presidents, ten ordinary members and seven volunteers. These diverse roles help to provide a comprehensive understanding of the perspectives and experiences of the different members, thus enhancing the representativeness and reliability of the findings of this research.

**Table 1:** Respondents information.

Number	Gender	Age	Identity	Number	Gender	Age	Identity
R1	Female	56	President	R13	Female	41	Member
R2	Male	38	President	R14	Male	42	Member
R3	Female	45	vice-president	R15	Male	43	Member
R4	Female	42	vice-president	R16	Male	48	Member
R5	Male	24	vice-president	R17	Female	27	Volunteer
R6	Female	25	vice-president	R18	Male	21	Volunteer
R7	Female	26	Member	R19	Female	28	Volunteer
R8	Female	37	Member	R20	Female	56	Volunteer
R9	Male	47	Member	R21	Male	21	Volunteer
R10	Female	45	Member	R22	Male	27	Volunteer
R11	Male	47	Member	R23	Male	23	Volunteer
R12	Female	39	Member				

### Ethical statement

The Medical Ethics Committee of the School of Basic Medicine (MECSBM) has approved this research. All respondents were informed of the content and purpose of the research. And this research has obtained written informed consent from all respondents.

## Results

### Social norms

Structural functionalism notes that social norms, as the basis of social order, are essential to the regular operation of society [38]. Social norms promote social solidarity by regulating the individual behavior of residents and facilitating interaction and collaboration among residents. Turkan pointed out that social norms are the source of social unity and consistency. It can be seen that social norms are essential in restraining the behavior of residents towards community cats. ACCA mainly constructs social norms from three aspects: behavioral norms, material modifications, and mechanism building. The behavioral norms provide a paradigm of behavior for residents to get along with community cats. The Community Animal Civility Convention issued by ACCA clearly states: ’For disputes related to community animals, the parties concerned shall negotiate and settle on their own, and if the negotiation fails, the relevant community organizations can mediate them,’ ’Do not destroy the equipment and supplies of community cats,’ ’Residents can feed cat food at fixed cat feeding points by themselves, and do not feed leftovers that affect hygiene,’ and so on. These norms help people understand their responsibilities and provide options for resolving disputes.

Material modification refers to constructing a suitable physical environment in public space to meet the basic needs of community cats and guide their behavior. It also promotes the orderly use of public space, guides people to handle problems related to community cats correctly, and reduces conflicts caused by community cats. As in ACCA, it has joined hands with property owners to build cat kennels and feeding facilities at suitable locations in the S community. At the same time, signs are erected near the activity areas of community cats, such as ’There may be community cats underneath the vehicle, please check the undercarriage of the vehicle before starting it.’ Establishing these infrastructures helps guide and regulate the behavior of residents and community cats. For community cats, these infrastructures provide more living space and resources and reduce the pressure of competition for food and territory. They do not have to roam around looking for food, reducing conflicts between them and residents. These infrastructures remind residents not to interfere with community cats and alert them to the presence of community cats so that both parties are not frightened. Meanwhile, clean and standardized feeding facilities also promote residents’ reasonable management of community cats, creating a friendly community environment. In order to further implement social norms, ACCA establishes an effective whistle-blowing mechanism and mediation mechanism. The whistle-blowing mechanism allows community residents to anonymously report behaviors involving abuse of community cats or other issues. The mediation mechanism means the association establishes a special committee responsible for receiving, investigating, and handling reported incidents. This resolves conflicts at the source and avoids escalation or inappropriate actions.

### Social trust

Community cat management requires the establishment of mutual trust. Social trust includes trust among residents and between residents and community cats. Trust between residents and community cats means that residents believe that community cats will not cause harm to the community and that community cats have a positive significance for the community. Residents need to have a certain degree of trust in community cats, be willing to coexist with them peacefully, and provide them with the necessary care and affection. Trust among residents means that residents need to believe that other residents and related organizations will contribute to the well-being of community cats and thus participate in managing community cats together.

The individual’s body perception ability is crucial for the trust between residents and community cats. Through body perception, individuals can observe the behaviors and expressions of community cats, thus obtaining more information about them. This perception is conducive to helping residents understand community cats’ needs, emotions, and intentions for more effective communication. At the same time, community cats can also interpret people’s intentions by observing their body postures and expressions. A relaxed body posture and facial expression can send a safe and friendly signal to community cats and promote positive interaction and communication. Therefore, it is crucial to guide residents to interact with community cats. Through the volunteer program, ACCA effectively guides residents’ physical perception and behavior when facing community cats. ACCA has implemented the Science Citizenship Program, which invites residents to participate in the scientific activities of observing and recording community cats. During the program, residents are taught interaction skills with community cats to deepen their understanding of and concern for individual animal life. In addition, the ACCA opened the community cat shelter and invited residents to interact with trained community cats. In this environment, residents can participate in various activities with the community cats, such as feeding, petting, and playing. During interaction with these community cats, residents will experience positive physiological responses. This is beneficial to cultivating residents’ care and respect for animals, which motivates them to treat community cats in a friendly manner and actively participate in animal protection and welfare matters in their daily lives.

For trust between residents, moral values are fundamental. On the one hand, moral concepts involve recognizing and practicing fairness and justice. In community cat management, residents must trust that other residents will follow the principles of fairness and justice and will not use community cat management for personal gain. Therefore, ACCA has established a transparent information disclosure and monitoring system. ACCA establishes an electronic profile of community cats, registering each community cat’s appearance, character, range of activities, and health status. This information helps residents understand the situation of community cats better and helps the community prevent and solve problems that may arise. At the same time, residents can monitor the association’s operation through complaints and suggestions.

On the other hand, residents with a sense of responsibility and empathy will be more willing to work together to manage community cats based on mutual trust. ACCA utilizes social media and online forums to disseminate information about animals in the community, share animal stories and photos to promote people’s empathy. The themes of the stories focus on three aspects: redemption, remembrance, and warmth. Redemption refers to the story of a community cat that is rescued and sheltered from a problematic situation and rediscovers the meaning and value of life. Remembrance refers to the expression of respect for the community cats by recalling the positive moments in their lives after their passing. Warmth refers to presenting the care and support of community residents for community cats and creating a warm and loving living environment for them. This emotional connection inspires a sense of moral responsibility in residents, prompting them to care for and respect community cats and to see them as living beings with rights and dignity. In short, these two types of trust reinforce each other. Trust among residents promotes trust in community cats. This is because when residents trust each other and jointly participate in protecting and managing community cats, they are more likely to trust community cats. At the same time, when residents trust them and recognize their existence, they are more likely to trust each other.

### Social network

To realize effective community governance and the maintenance of public interests, the participation of community residents is an essential factor. Only by mobilizing residents’ enthusiasm, activating community participation, and forming a stable relationship network can the community governance system constructed with the participation of multiple actors have lasting vitality. The relationship network provides resources and information for community management and promotes cooperation and coordination among residents. On the one hand, ACCA has built an online community that enables residents to connect with residents and vets. This community support network allows residents to share their experiences of interacting with community cats with each other. It provides timely assistance to residents who encounter problems related to community cats. More importantly, residents can form collective moral pressure through mutual supervision, prompting residents to consciously assume the responsibility of caring for and managing community cats.

On the other hand, ACCA established a professional community animal rescue team responsible for providing professional feeding and medical assistance to community cats. This ensures the safety of food and medicine and eliminates indiscriminate feeding by individual residents. Through forming a relationship network, the association has achieved the organizational management of community cats. Compared to individual rescue, the advantages of organizational management are resource integration, long-term planning, and information sharing. Resource integration means that ACCA can more easily obtain support from the government and enterprises, thus obtaining more resources. Long-term planning means the organization can carry out longer-term planning and implement more structured management programs. ACCA sets long-term goals, such as controlling the number of community cats and improving their quality of life, and gradually achieves these goals. Information sharing means ACCA usually has a broader network and can cooperate with other related organizations, experts, or volunteers. Through information sharing, the association can gain more experience and promote different actors to work together to solve various problems in community cat management.

## Discussion

By guiding the behavior of the residents, ACCA has succeeded in establishing a social network linked to the care and management of community cats. This link connects community residents of different statuses and activates the social capital of the local community. Many empirical studies have demonstrated social capital’s critical role in various aspects of community development, covering various areas such as economic development, community governance, race relations, and democratic participation [39, 40]. The above results found that social norms, trust, and relational networks are essential for integrating community cats into community governance. Social norms constrain community cats’ breeding and activity range and reduce residents’ unfriendly treatment of community cats. Social trust is conducive to reducing the cost of community governance and helps promote residents’ cooperation and joint participation in community cat management. With social norms and trust established, community residents formed a community relationship network centered on ’community cats’. Community relationship networks are essential carriers of social capital. The closely-knit relationship network can integrate the community into a complex community with emotional ties and interests intertwined.

The key to the community relationship network centered on the ’community cat’ is to extend the care of the community cat to other residents in the relationship network. When residents participate in the care of the community cats, they are not only concerned about the well-being of the community cats but also begin to realize the value and importance of each life. That is, caring for community cats can develop empathy, responsibility, and a caring spirit. This can teach residents to be responsible for the weak and other living beings and make them more aware of their strong connection to the natural world. This shift in awareness further inspires residents to care for other members of the community. In this paper, we summarize this phenomenon as ’care extension’, which means that by taking care of community cats, residents extend their care to other members of the community.

**Fig 5:**
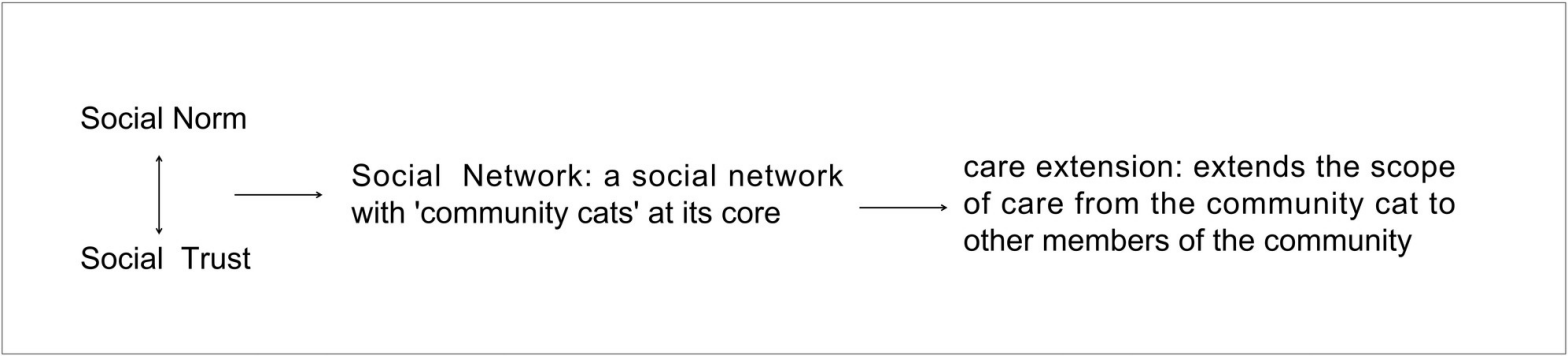
Social mechanisms for care extension.

This care extension is a chain reaction triggered by the care and management of community cats (Fig 5). In participating in the care and management of community cats, residents form a cooperative network. This not only enhances social trust among residents, but also promotes mutual help. For example, by encouraging the elderly and lonely residents to participate in the care of community cats, it not only provides them with emotional companionship, but also empowers them with more social roles and responsibilities, reduces loneliness and enhances the quality of life. This helps these groups reintegrate into community life and enhances their sense of belonging. In addition, the cooperative network established in participating in the care and management of community cats will be extended to other aspects of community life. This enables residents to reach consensus and collaborate with each other more efficiently on other community affairs and projects. For example, when organizing community events, the network quickly mobilizes residents to volunteer. In the face of the closure policy during the COVID-19 pandemic, the network facilitated residents helping each other to take care of elderly family members or companion animals. The realization of the care extension means that community cats are successfully integrated into the community governance. It marks a step towards a more inclusive and caring community. On the one hand, the community has established a community cat governance system centered on the ACCA. All actors can fully utilize their respective strengths to ensure the safe and healthy life of community cats. On the other hand, the cohesion and mutual help spirit among community residents has been further stimulated through the success stories of community cats. People have established closer community ties. This not only realizes the harmonious coexistence of residents and community cats, but also promotes the formation of a more friendly community.

## Conclusion

This research found that residents were able to accept community cats as city dwellers and integrate them into urban community governance. This means that residents and community cats have formed a neighborhood relationship beyond the ecological one. This relationship has a rich emotional, cultural, and social connection between people and community cats. The community cat is no longer just a biological resource to be managed and utilized but an actor with an agency that can interact with people.

This research views community governance as a social process involving multiple species. Based on the analytical framework of ’norms, trust, and networks’ from social capital theory, the research finds that social norms and social responsibility activate the social capital of the community, forming a social network with community cats as the core actors, and then realizing the care extension. In caring for and managing the community cats, the residents take the initiative to extend their care to other community members. The realization of the care extension means that the community cat has been successfully integrated into the community governance. In conclusion, non-human animals have begun to enter the field of sociological research in a completely new posture. Researchers should focus on a more harmonious and balanced model of human and non-human animals living together. By treating non-human animals as actors in social governance, we can better recognize their interactions with human society. This allows us to see the impact of non-human animals on our lives, values, and social structures. This emphasis on non-human animals also pushes us to rethink the applicability and limitations of sociological concepts in order to more accurately explain and understand the complexity of human societies and deepen our knowledge and understanding of human beings.
